# The Effect of Blade Alignment on Kinematics and Plantar Pressure during the Execution of Goaltender-Specific Movement Patterns: A Case Study

**DOI:** 10.3390/sports10060096

**Published:** 2022-06-20

**Authors:** Colin Dunne, Michael Holmes, Kelly Lockwood

**Affiliations:** Department of Kinesiology, Faculty of Applied Health Sciences, Brock University, 1812 Sir Isaac Brock Way, St. Catharines, ON L2S 3A1, Canada; mholmes2@brocku.ca (M.H.); klockwood@brocku.ca (K.L.)

**Keywords:** biomechanics, performance, hockey, goaltender, skates, blades, alignment

## Abstract

Innovations in material properties of goaltender skates have improved the protective characteristics of the boot, leading to redesign of the blade holder to resemble players’ holders. The redesigned blade holder introduces the ability to customize blade alignment, which may grant a performance advantage. We investigated the effect of blade alignment on kinematics and plantar pressure during the execution of two different goaltender-specific movement patterns: (1) the butterfly drop to recovery and (2) the lateral butterfly slide to recovery. The main objective of this study was to investigate the effect of three blade alignment conditions. The secondary objective was to compare two neutral alignment conditions, which was facilitated by studying the effects of two different holders on kinematics and plantar pressure during two goaltender-specific techniques. A male goaltender with professional experience completed an A–B–A design, executing five trials of A, B, and A for both movements with each blade alignment condition (*n* = 30 per collection, *n* = 90 overall) on synthetic ice in a controlled lab environment. Blade alignment conditions were defined by the alignment of the blade holder on the boot and the type of blade holder. Kinematic and plantar pressure data were collected simultaneously using 3D motion capture and in-skate pressure insoles, respectively. Increased butterfly drop velocity (2.07 ± 0.09 m/s) and peak plantar pressure (77.19 ± 2.67 psi) were revealed when executing the butterfly drop with medial alignment. This work suggests medial blade alignment may enable the goaltender to drop into the butterfly position faster, potentially increasing the likelihood of making a save.

## 1. Introduction

In the sport of ice hockey, goaltender equipment is critical to both the athlete’s protection and performance. Goaltender-specific equipment was first introduced in the late 1800s with leg pads and a widened stick in 1893 [1]. The first goaltender-specific skates were manufactured in 1908 and evolved over time to consist of the boot, the cowling, and the blade runner [1]. The boot was originally made from soft and supple leather that provided limited protection, and, as such, a plastic form called a cowling was wrapped around the lower portion of the boot to provide additional protection as well as to serve as a rigid interface between the boot and blade [2]. Consistent with current innovations in the development of player skates, the material properties of goaltenders’ skate boots have evolved from leather to synthetic materials, carbon fibers, and resins with reinforced toecaps to improve protection, structure, and durability [3,4]. Stronger, stiffer, and more protective boots eliminated the need for the cowling, and, as such, the cowling has been replaced by a blade holder that resembles the design of a forward or defense player blade holder. 

Blade alignment describes the positioning of the holder and blade on the skate boot. At more advanced levels of play and athletic ability, it is common practice to customize blade alignment of a player’s skates to suit individual anatomical configurations and preferences. Specific to goaltenders, alignment was handcuffed by the design and fit of the cowling, as it prevented lateral movement of the blade runner in relation to the boot. Redesign of the goaltender blade holder presents the opportunity to move the blade holder medially or laterally to manipulate skate blade alignment. However, no published research has investigated the potential impact of hockey skate blade alignment positioning on athlete performance, either hockey players or goaltenders. A similar concept of blade alignment was investigated for speed skates, where research has investigated the impact of modifying the alignment of the pivot point of the klapskate hinge (which allows the boot to rotate relative to the blade holder) based on mechanical changes of hips, knees, and ankle joints of speed skaters [5,6]. Alignment of the klapskate hinge on a speed skate in an anterior direction increased hip and knee ranges of motion and peak angular velocities and a decrease in angular velocity of the ankle [5,6], providing justification that blade alignment has an effect on relevant speed skating metrics that may impact performance.

The purpose of this study was to investigate the effect of blade alignment on kinematics and plantar pressure during the execution of two different goaltender-specific movement patterns: (1) the butterfly drop to recovery and (2) the lateral butterfly slide to recovery. The study addressed two research objectives in an attempt to understand the contribution of blade alignment to the execution of two variations of the butterfly technique. The main objective of this study was to investigate the effect of three blade alignment conditions (alignment neutral (AN), alignment lateral (AL), and alignment medial (AM)) on kinematics and plantar pressure during two goaltender-specific techniques. The secondary objective was to compare two neutral alignment conditions, facilitated by two different holder types—Bauer Vertexx cowling (alignment neutral cowling (ANC)) and True Hockey blade holder (alignment neutral (AN))—on kinematics and plantar pressure.

## 2. Materials and Methods

### 2.1. Participant

For this case study, a male goaltender (27 years, 193 cm, 79 kg) with professional hockey experience served as a single participant. A single-participant design was implemented to limit variability introduced by differences in technique, styles of play, and individual anatomical alignments (i.e., neutral, pronated, supinated) across multiple participants. It is important to note that this case report is a single-participant design due to the required customization of the skates and insoles (as described below) required to facilitate our blade alignment research. Eligibility criteria included currently playing competitive hockey, injury free, and self-identified that the butterfly technique was their preferred save technique. The participant wore True Pro Custom skate boots (True Temper Sports Inc., Memphis, TN, USA), True holders/Bauer Vertexx cowlings (Bauer Hockey, Exeter, NH, USA) and 4 mm Step Steel blades. Their own protective equipment including glove, blocker, stick, knee pads, leg pads, and form-fitting clothes (pants and shirt) typically used for game play were also worn. Ethical clearance was obtained from the Office of Research Ethics Board of Brock University (File #18-096).

### 2.2. Study Design

To allow a more robust analysis of the single-participant case study, an A–B–A research design was conducted to investigate the effect of different blade alignment conditions during the execution of two goaltender-specific movement patterns: (i) butterfly drop to recovery and (ii) lateral butterfly slide to recovery. Data collection was scheduled one day per week for three weeks, with each session introducing a different blade alignment condition (B). 

### 2.3. Alignment Conditions

**Figure 1:**
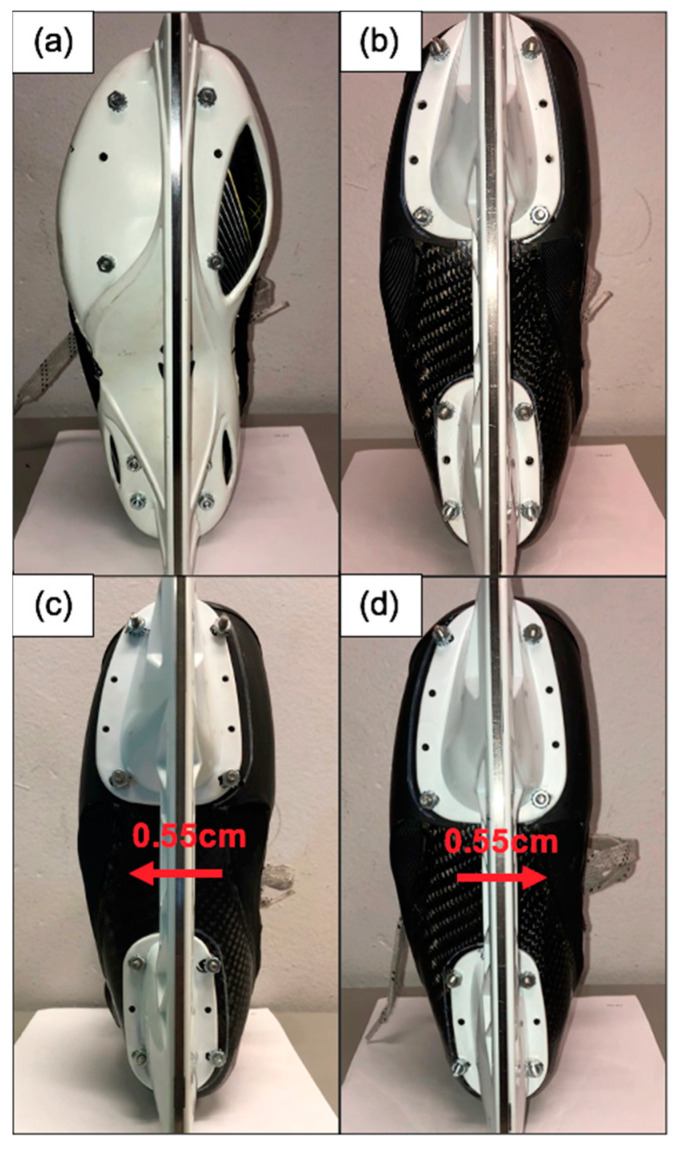
Blade alignment conditions: (**a**) Alignment neutral cowling (ANC), (**b**) Alignment neutral (AN), (**c**) Alignment lateral (AL), (**d**) Alignment medial (AM). Note: The modified blade holders positioned the blade runner an equal distance (0.55 cm) from neutral based on the maximal achievable distance from neutral that could be facilitated by the True blade holders.

Blade alignment conditions used for the study were defined by (i) the alignment of the blade holder on the boot and (ii) the style of blade holder. Three pairs of True Hockey blade holders were retrofitted with a slotted design to facilitate comparison of the three different alignment conditions (AN, AL, and AM) as described below (Figure 1):

1. Alignment neutral (AN): centered (neutral) blade alignment.
2. Alignment lateral (AL): blade positioned 0.55 cm lateral from the centre of the boot.
3. Alignment medial (AM): blade positioned 0.55 cm medial from the centre of the boot.

**Figure 2:**
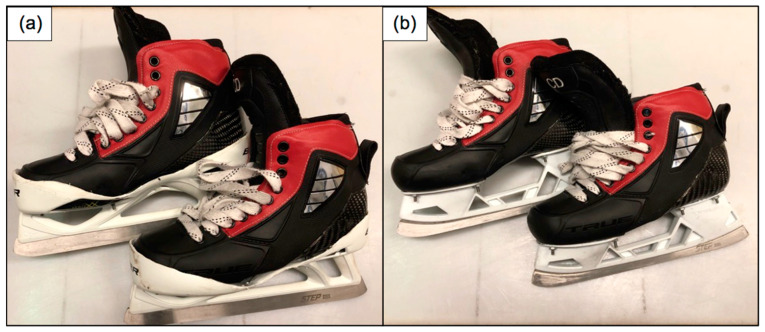
True Pro-Custom skate boot with (**a**) Bauer Vertexx cowlings, (**b**) True holders.

For the style of blade holder, neutral blade alignment facilitated by a Bauer Vertexx cowling (ANC) (Figure 2a) was compared to neutral alignment facilitated by the True Hockey blade holder (AN) (Figure 2b). The ANC session was utilized as the baseline condition in the A–B–A design for the purpose of investigating potential holder type effects.

The same True Pro Custom skate boots were worn for all sessions. Blade runner height, width, and sharpening were consistent; radius of contour was 9.0 m, radius of hollow was 1.5 cm, and pitch was neutral. Blades were sharpened prior to each testing session.

### 2.4. Goaltender-Specific Movement Patterns

**Figure 3:**
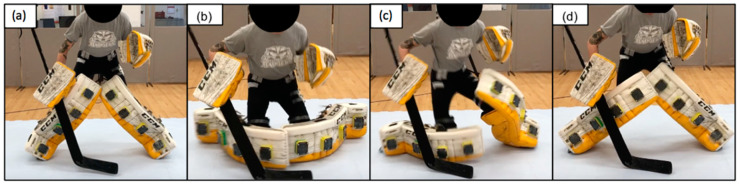
Butterfly drop to recovery consisting of (**a**,**b**) phase one, (**c**) phase two, and (**d**) phase three.

The goaltender-specific movement patterns were focused on movements involving the butterfly as the butterfly has been revealed to be the most common save technique used by goaltenders at the NHL level at 34 ± 6 times per game [7]. A detailed technical description of the goaltender-specific movement patterns used for analyses is as follows:

Butterfly drop to recovery (movement one) consisted of three phases (Figure 3): Phase one, the butterfly drop, whereby the goaltender started in an upright ready stance and executed a butterfly drop by dropping to both knees with the medial aspect of both lower legs pads parallel and in contact with the ice. Phase two, the left leg butterfly recovery, whereby the left leg transitioned from the butterfly position back to the ready stance. Phase three, right leg butterfly recovery, whereby the right leg transitioned from the butterfly position to the ready stance. The entire movement was concluded when the goaltender was back in the ready stance. 

**Figure 4:**
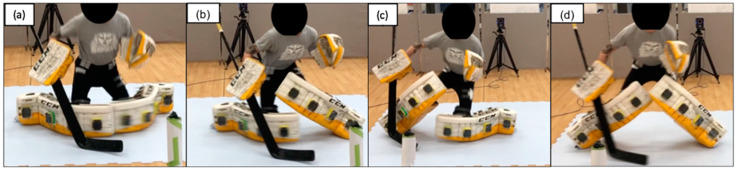
Lateral butterfly slide to recovery consisting of (**a**) phase one, (**b**) phase two, (**c**) phase three, and (**d**) phase four.

Lateral butterfly slide to recovery (movement two) consisted of four phases (Figure 4): Phase one, the butterfly drop, defined above. Phase two, the lateral butterfly slide, whereby the goaltender slid to his right via a push with the medial edge of the left blade runner. Phase three, right leg butterfly recovery, whereby the right leg transitioned from the butterfly position to the ready stance. Phase four, left leg butterfly recovery, whereby the left leg transitioned from the butterfly position back to the ready stance. The entire movement was concluded when the goaltender was back in the ready stance. Phase one of movement one and movement two involved the same technique. 

### 2.5. Experimental Protocol

All trials were executed in a controlled laboratory environment on a 5.7 m^2^ synthetic ice sheet (xHockeyProducts^TM^, Green Brook Township, USA). Previous research comparing real ice and synthetic ice has revealed minimal differences in forward skating kinetics and kinematics of hockey players [8], suggesting it is a valid alternative for lab-based research. A familiarization period was consistent across each blade alignment, including a five-minute warm-up consisting of self-selected patterns of goaltending specific drills including shuffle, T-push, butterfly, lateral butterfly slides, and recoveries. Five trials were executed on each of the baseline–condition–baseline blade alignment conditions for both movements (*n* = 30 trials per collection day, *n* = 90 trials overall). Following each trial, the participant was required to confirm technical adequacy and effort. Technically inadequate trials were repeated.

### 2.6. Data Collection

**Figure 5:**
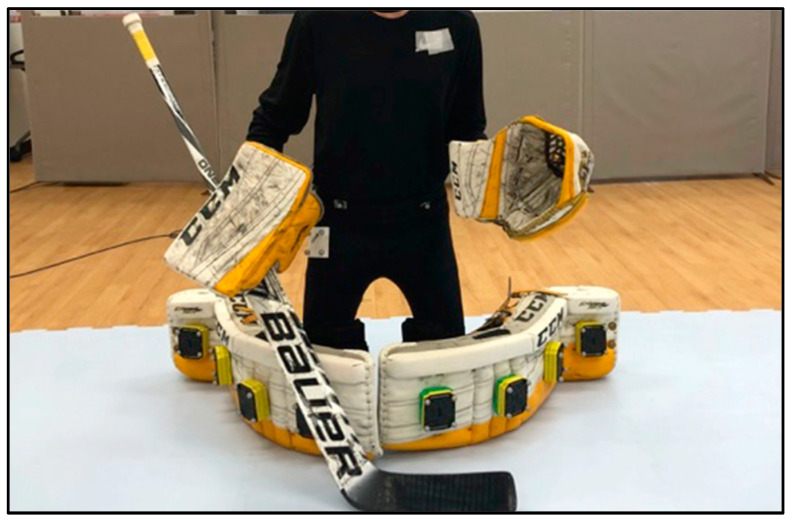
Three-dimensional marker set and synthetic ice.

Kinematic and plantar pressure data were collected simultaneously for all trials. Three-dimensional kinematics were collected using a ten-camera Vicon motion capture system sampling at 330 Hz (Vicon^TM^, Oxford, UK). The Vicon motion capture system was calibrated prior to each data collection. A forty-two reflective marker configuration adapted from a goaltender-specific marker set designed to decrease interference due to goaltender equipment [9] (Figure 5) was used. Ten markers were placed on the body, including left and right locations of the anterior superior iliac spine, posterior superior iliac spine, heel, and a four-marker rigid cluster on the left lateral thigh. Thirty-two markers were placed on the leg pads, including a four-marker rigid cluster on the upper thigh region, knee roll region, shank region, and toe region for both pads. The global coordinate system was defined as follows: Z (vertical), X (medial/lateral), and Y (anterior/posterior).

Plantar pressure data were collected using a wireless portable plantar pressure distribution insole system (LogR^TM^, Orpyx^®^ Medical Technologies Inc. Calgary, Canada) connected via Bluetooth to an iOS device. Each insole consisted of eight plantar pressure sensors sampling at 100 Hz. The LogR^TM^ insoles were calibrated by Orpyx^®^ Medical Technologies Inc. prior to data collection. Importantly, and in addition to the custom skate used in this case study, the insoles were customized to fit the participant’s in-skate footbed dimensions. Insoles were inserted into the skates, and the participant tied their skates similarly to game conditions. The lightweight insole data logger was tethered through the laces of the boot and secured to the anterior aspect of the skate boot to avoid interference with the pad. Prior to each blade alignment condition, insoles were zeroed (tared) as the participant held his legs (with pads strapped on) in the air with no body weight applied. 

### 2.7. Data Processing

#### 2.7.1. Kinematics

Kinematics were processed independently using Visual3D (C-Motion Inc. v6 Professional, Germantown, MD, USA). Event markers were defined for the phases of the goaltender-specific movement patterns based on the top and most lateral marker on the left and right upper thigh region rigid cluster. Kinematic outcome measures included:

I. Butterfly drop velocity (BDV(m/s)): displacement of the markers in the *Z*-axis was used to calculate average velocity from the ready stance until the leg pads were parallel to the ice in the butterfly position.
II. Left leg recovery velocity (LLRV(m/s)): displacement of the markers in the *Z*-axis was used to calculate average velocity from the onset of recovery movement of the left leg pad back to the ready stance.
III. Right leg recovery velocity (RLRV(m/s)): displacement of the markers in the *Z*-axis was used to calculate average velocity from the onset of recovery movement of the right leg pad back to the ready stance.
IV. Lateral butterfly slide velocity (LBSV(m/s)): displacement of the markers in the *X*-axis was used to calculate average velocity from the onset of the lateral push until the onset of the right leg butterfly recovery (only collected during movement two).
V. Butterfly width (BW(m)): displacement in the X and *Y*-axis between the top toe markers on the left and right pads when in the butterfly position (only collected during movement one).

#### 2.7.2. Plantar Pressure

Raw plantar pressure data (psi) were exported into Microsoft Excel (v16.33) (Redmond, USA) and event markers were defined for the phases of the goaltender-specific movement patterns based on the onset of pressure on the corresponding insole. In-skate peak plantar pressure (PPP (psi)) was calculated and defined as the peak plantar pressure occurring during the individual movement phases of the goaltender-specific movement pattern being performed. Due to the bilateral nature of the butterfly drop phase of both movements, PPP was a summation of left and right insole data. PPP collected during all other phases of both movements were analyzed unilaterally, for example, during the left leg recovery the left insole was analyzed.

### 2.8. Descriptive Analysis 

Data calculated included descriptive analysis, including mean and standard deviation (SD) (mean ± SD) for all kinematic and plantar pressure variables. To address the main purpose of this study, kinematics (BDV, LLRV, RLRV, LBSV, BW) and peak plantar pressures (PPPs) were compared across three blade alignment conditions (AN × AL × AM) during the execution of each phase of both movements. To address the secondary purpose of this study, kinematics (BDV, LLRV, RLRV, LBSV, BW) and peak plantar pressures (PPPs) were compared between two holder types (ANC × AN) during the execution of each phase of both movements.

## 3. Results

### 3.1. Blade Alignment Conditions

**Figure 6:**
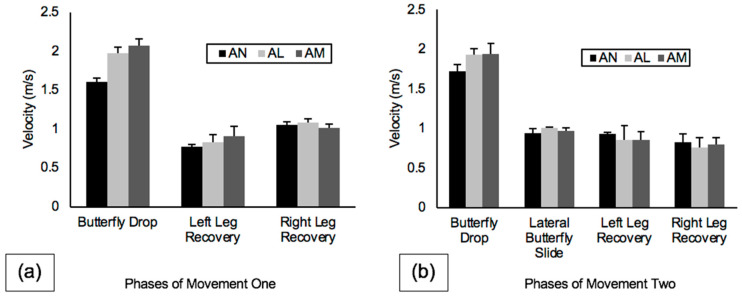
Velocity (m/s) across alignment conditions (AN, AL, AM) for all phases of (**a**) movement one and (**b**) movement two. Note: Error bars represent standard deviations between trials.

Velocity data for the three blade alignment conditions for movement one and movement two are shown in Figure 6. During movement one, BDV was fastest on AM (2.07 ± 0.09 m/s), LLRV was also fastest on AM (0.91 ± 0.12 m/s), RLRV was fastest on AL (1.08 ± 0.05 m/s), and BW was greatest on AM (1.40 ± 0.01 m). During the execution of movement two, BDV was again fastest on AM (1.94 ± 0.13 m/s), LBSV was fastest on AL (1.01 ± 0.01 m/s), and both LLRV and RLRV were fastest on AN (left, 0.93 ± 0.02 m/s, right, 0.83 ± 0.10 m/s).

**Figure 7:**
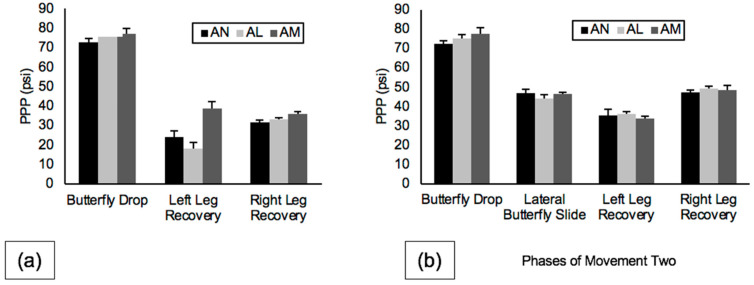
PPP (psi) across alignment conditions (AN, AL, AM) for all phases of (**a**) movement one and (**b**) movement two. Note: Error bars represent standard deviations between trials.

Peak plantar pressure data for the three blade alignment conditions for movement one and movement two are shown in Figure 7. During movement one, butterfly drop PPP was highest on AM (77.19 ± 2.67 psi), and both left leg recovery PPP and right leg recovery PPP were also highest on AM (left, 38.75 ± 3.60 psi, right, 35.88 ± 1.40 psi). During movement two, butterfly drop PPP was again highest on AM (77.69 ± 2.98 psi), lateral butterfly slide PPP was highest on AN (46.75 ± 2.22 psi), and both left leg recovery PPP and right leg recovery PPP were highest on AL (left, 36.00 ± 1.22 psi, right, 49.22 ± 1.16 psi).

### 3.2. Holder Types

**Figure 8:**
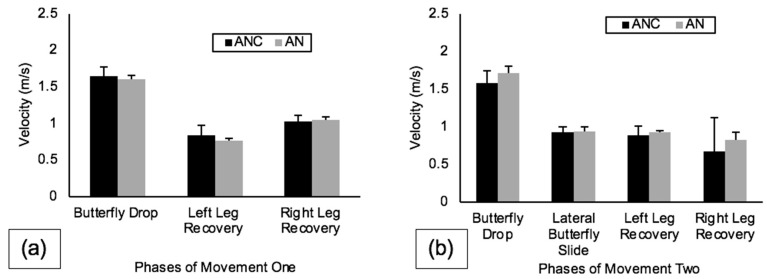
Velocity (m/s) between neutral alignments (ANC, AN) for all phases of (**a**) movement one and (**b**) movement two. Note: Error bars represent standard deviations between trials.

Velocity data for the two holder conditions for movement one and movement two are shown in Figure 8. During movement one, BDV was fastest on ANC (1.65 ± 0.12 m/s), LLRV was also fastest on ANC (0.84 ± 0.14 m/s), RLRV was fastest on AN (1.08 ± 0.04 m/s), and BW was greatest on AM (1.39 ± 0.02 m). During movement two, BDV was fastest on AN (1.72 ± 0.09 m/s), LBSV was also fastest on AN (0.94 ± 0.06 m/s), and both LLRV and RLRV were fastest on AN (left, 0.93 ± 0.02 m/s, right, 0.83 ± 0.10 m/s).

**Figure 9:**
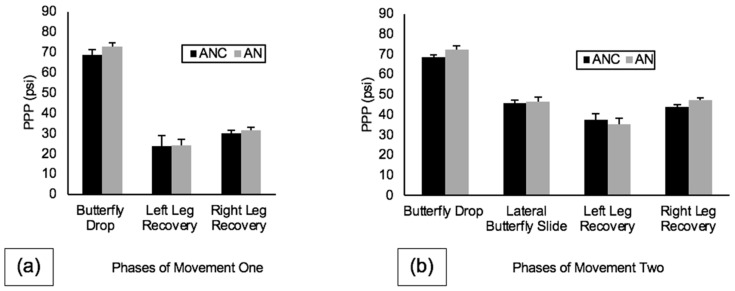
PPP (psi) for neutral alignments (ANC, AN) for all phases of (**a**) movement one and (**b**) movement two. Note: Error bars represent standard deviations between trials.

Peak plantar pressure data between the three blade alignment conditions for movement one and movement two are shown in Figure 9. During movement one, butterfly drop PPP was highest on AN (72.62 ± 2.21 psi), and both left leg recovery PPP and right leg recovery PPP were also highest on AN (24.06 ± 3.10 psi and 31.73 ± 1.24 psi, respectively). During movement two, butterfly drop PPP was again highest on AN (72.39 ± 1.76 psi), lateral butterfly slide PPP was highest on AN (46.75 ± 2.22 psi), left leg recovery PPP was highest on ANC (37.52 ± 3.11 psi), and right leg recovery PPP was highest on AN (47.36 ± 1.22 psi). 

## 4. Discussion

Replacement of cowlings with player-like goaltender blade holders presented the opportunity to investigate the effect of blade alignment on kinematics and plantar pressure during two different goaltender-specific movement patterns, the butterfly drop to recovery (movement one in this work) and the butterfly drop slide to recovery (movement two in this work). Outcomes of this case study suggest that blade alignment can change both kinematics and plantar pressures during these two movements. Faster movements and higher peak plantar pressures were revealed during the butterfly drop, recoveries, and lateral butterfly slide movements, which may promote more optimal positioning to potentially increase the likelihood of stopping the puck. Beyond the impact on the goaltender’s technical performance, the practical application of the research outcomes provides further insight and direction for hockey equipment manufacturers and equipment managers in the design and customization of equipment. 

The effect of blade alignment was investigated by comparing the kinematics and plantar pressures collected during each phase of movements one and two performed on three different blade alignment conditions (AN, AL, AM). BDV for movements one and two was fastest when performed on the AM blade alignment. The first metatarsal sensor registered the highest individual peak plantar pressure during the butterfly drop phase of both movements, and, considering that AM blade alignment positioned the blade holder on the medial edge of the boot under the first metatarsal, this may have facilitated increased butterfly drop velocity. Another potential reason for the fastest butterfly drop on AM may be due to the increased attack angle. Attack angle is defined as the angle at which the blade can remain in contact with the ice before the medial edge of the skate boot contacts the ice, causing the blade to lose contact with the ice [10]. An increase in attack angle allows the blade to remain in contact with the ice through a greater range of motion and therefore generate force for a longer period of time. Differences in butterfly width were negligible, as the largest mean difference was between AM and AN at 0.009 mm.

PPP collected during the butterfly drop phase of movements one and two and for both recovery phases (LLRV, RLRV) of movement one was greatest with AM blade alignment. Similar to the kinematics, a possible explanation could be that these phases of movement are initiated by the medial edge of the skate blades driving into the ice, with plantar pressure being predominantly driven through the athlete’s first metatarsal. Therefore, positioning the blade closer to the athlete’s region of highest PPP may have contributed to AM having greater PPP, as force was exerted directly through the blade and into the ice rather than on an angle to the blade. Alternatively, greater PPP for the AM condition may be due to the increased attack angle, allowing the goaltender to generate force at a smaller angle with the ice compared to AN and AL. Faster LLRV and left leg recovery PPP were also revealed on AM during movement one. RLRV and right leg recovery PPP did not follow similar trends.

BDV and PPP of the butterfly drop phase of both movements were highest on AM, consistent with research investigating the contribution of equipment to performance for different soccer shoes [11]. Findings demonstrated that shoes with faster sprint performance were associated with higher summed peak pressure under the first metatarsal head and medial heel. Greater force on soccer shoe studs increased traction, resulting in faster locomotion [11]. Within sport-specific research, increased peak plantar pressure in certain footwear conditions has improved performance of specific movements in an array of sports, including soccer [11], fencing [12], field hockey [13], and running [14].

The effect of holder type was investigated by comparing the kinematics and plantar pressures collected during each phase of movements one and two on two different neutrally aligned holder types (ANC and AN). The expectation was confirmation that neutral alignment was neutral, independent of the type of holder being used to position the blade on the boot. Very minor and inconsistent differences in kinematics and PPP were revealed across the phases of both movement patterns between the ANC and AN holders. These results provide evidence that very little difference exists between holders when both are positioned in neutral alignment. Butterfly drop PPP during the butterfly drop phase of movement two was the only exception, with AN having a mean 3.79 psi higher than ANC. This difference resulted in AN having a 0.12 m/s faster mean for BDV for the same phase, similar to the results of the data found on AM for the butterfly drop phase of movements one and two. This increase in PPP and BDV on AN compared to ANC for the butterfly drop may also be explained by an increased attack angle of AN compared to ANC due to the bulk of the plastic cowling on the medial aspect of the boot in the ANC holder [10].

Research outcomes primarily support the contribution of blade alignment to goaltender-specific movement patterns, specifically BDV. This is consistent with research investigating the contribution of equipment to performance in ice hockey goaltenders [9,15,16]. Different goaltender pad leg channels revealed differences in peak butterfly drop velocity, specifically a flex-tight leg pad channel [9], and differences in butterfly width measures (0.22 cm) [15]. New compared to broken-in goaltender pads decreased internal hip rotation range of motion [16]. Consistent with research in speed skating [5,6], blade alignment was revealed to affect hockey goaltender kinematics.

The primary role of a goaltender is to stop the puck from going in the net, thus preventing the opposing team from scoring. This is facilitated by moving into position as quickly as possible in order to make saves. The most common save technique used by goaltenders is the butterfly, performed 34 ± 6 times per game at the NHL level [7]. Enhancing the goaltender’s save-positioning ability has the potential to prevent the puck from entering the net. In isolation or outside the context of game performance, the differences in the results may seem relatively minimal; however, when interpreted in the context of the fast-paced sport of hockey, the outcomes may have significant practical applications. For example, based on the mean vertical displacement from the ready stance to butterfly positioning for goaltenders (0.49 m) for all trials and mean shot velocities by college level players (30.6 m/s) [17], the goaltender could achieve the butterfly positioning in time for the puck to make contact with them for a shot from: AN-9.00 m, AL-7.65 m, or AM-7.47 m. Therefore, in a scenario where the goaltender is using AM blade alignment, the goaltender can perform the butterfly drop into butterfly positioning for a shot 1.53 m closer than for the AN blade alignment. Providing the goaltender the ability to get into position for a larger percentage of total shot scenarios is a major advantage in a hockey game, especially considering the offensive zone of the rink is only 19.51 m long [18]. 

Within the hockey industry, blade alignment customization is available to player skates; however, it is typically used at the elite levels of play, where equipment managers are employed. Expertise governs this practice, and no instrumentation provides information for ideal blade alignment per individual. When a goaltender buys skates, the concept of blade alignment was traditionally not an option, and furthermore, there was no expertise to inform this practice. The ability to manipulate goaltender skate blade alignment became an option only after the cowling had been removed, and the new holder design was introduced in 2015 [3,4]. Although manufacturers do not typically customize skates for the masses, they may want to consider standardizing an alignment that suits the greatest number of goaltenders or engineer a slotted blade system that allows goaltenders to manipulate their blade alignment to suit their own preference. 

The case study design of this study was selected to limit variability introduced by differences in technique, styles of play, and individual anatomical alignments (i.e., neutral, pronated, supinated) across multiple participants. Along with this, the financial and technical nature of the equipment and instrumentation limited the number of participants. Skate boots were built to custom fit the participant, and the insoles were custom fit to the footbed of the skate boots. The case study design limits the ability to make broad conclusions in regard to goaltender blade alignment setup; however, it provides a foundation to support a larger investigation.

## 5. Conclusions

Traditionally, at the elite level, manufacturers do not mount holders on skates prior to shipping. It is the responsibility of the equipment manager to mount the blade holder on the boot, taking into account the individual athlete’s anatomical configuration and preferences. Outcomes of this study may not explicitly inform the athlete or equipment manager what blade alignment is best-suited for all goaltenders; however, it provides support for options other than the traditional neutral alignment. For practical applicability, results of this study may inform individual goaltender and equipment technician decisions on blade alignment that has the potential to enhance performance. The results of this study may also elicit future research to investigate the relationship between kinematics and peak plantar pressure during goaltender-specific movement patterns. In summary, results of the study add to the small body of research that focuses on the contribution of equipment to technique and, specifically, customizing alignment to potentially enhance goaltender-specific movement patterns.

## Data Availability

Not applicable.

## References

[1] Windsor Hockey Heritage Society—The Birthplace of Hockey: Ice Hockey Equipment 1863–1899 Evolution, 1900–1912 Evolution, 1912–1960 Evolution. http://www.birthplaceofhockey.com/evolution/.

[2] Bourque R. (1982). Goaler skate boot. U.S. Patent.

[3] Van Horne S. (2016). Protective goalie skate boot body with integral blade mounting channel. Patent.

[4] Dubois S., Desrochers C.A., Leblanc A., Harvey G., Seguin A. (2020). Skate for a hockey goalkeeper. Patent.

[5] Houdijk H., de Koning J.J., de Groot G., Bobbert M.F. (2002). How the klapskate hinge position affects push-off mechanics in speed skating. J. Appl. Biomech..

[6] Van Horne S., Stefanyshyn D. (2005). Potential method of optimizing the klapskate hinge position in speed skating. J. Appl. Biomech..

[7] Bell J.G., Snydmiller G.D., Game A.B. (2008). An Investigation of the Type and Frequency of Movement Patterns of National Hockey League Goaltenders. Int. J. Sports Physiol. Perform..

[8] Stidwell J.T., Pearsall D., Turcotte R. (2010). Comparison of skating kinetics and kinematics on ice and on a synthetic surface. Sports Biomech..

[9] Frayne R.J., Dickey J.P. (2017). Quantifying Ice Hockey Goaltender Leg Pad Kinematics and the Effect That Different Leg Pad Styles Have on Performance. Sports Eng..

[10] Woodley K. Unmasked: New Skates Giving Goalies an Edge. https://www.nhl.com/news/unmasked-new-skates-giving-goalies-an-edge/c-787521.

[11] Hennig E.M. (2014). Plantar Pressure Measurements for the Evaluation of Shoe Comfort, Overuse Injuries and Performance in Soccer. Footwear Sci..

[12] Geil M.D. (2002). The Role of Footwear on Kinematics and Plantar Foot Pressure in Fencing. J. Appl. Biomech..

[13] Lee J.Y., Lee J.S. (2012). Comparative Analysis of Plantar Pressure between Skilled and Unskilled Players during Hockey Penalty Stroke. KJSB.

[14] Nagahara R., Mizutani M., Matsuo A., Kanehisa H., Fukunaga T. (2018). Association of Sprint Performance with Ground Reaction Forces during Acceleration and Maximal Speed Phases in a Single Sprint. J. Appl. Biomech..

[15] Frayne R.J., Kelleher L.K., Wegscheider P.K., Dickey J.P. (2015). Development and Verification of a Protocol to Quantify Hip Joint Kinematics an Evaluation of Ice Hockey Goaltender Pads on Hip Motion. Am. J. Sports Med..

[16] Wijdicks C.A., Philippon M.J., Civitarese D.M., LaPrade R.F. (2014). A Mandated Change in Goalie Pad Width Has No Effect on Ice Hockey Goaltender Hip Kinematics. Clin. J. Sport Med..

[17] Wu T.C., Pearsall D., Hodges A., Turcotte R., Lefebvre R., Montgomery D., Bateni H. (2003). The performance of the ice hockey slap and wrist shots: The effects of stick construction and player skill. Sports Eng..

[18] USA Hockey Appendix IV-Official Rink Diagrams. https://www.usahockeyrulebook.com/page/show/1018530-appendix-iv-official-rink-diagrams.

